# 41896 Surveying knowledge of quality of dementia care among Latino caregivers

**DOI:** 10.1017/cts.2021.620

**Published:** 2021-03-31

**Authors:** Michelle S. Keller, Sara McCleskey, Allison Mays, Catherine Sarkisian

**Affiliations:** 1 Cedars-Sinai Medical Center; 2 UCLA

## Abstract

ABSTRACT IMPACT: This qualitative study describes health system barriers to high-quality care for Latino older adults with Alzheimer’s Disease and Related Dementias OBJECTIVES/GOALS: Compared to non-Latino Whites, Latino older adults are more likely to receive low-quality dementia care such as high-risk medications or services. Caregivers play a critical role in managing medical care for persons with dementia (PWD). Yet little is known about the perceptions and knowledge of dementia quality of care among Latino caregivers of PWD. METHODS/STUDY POPULATION: We used a qualitative research design and conducted interviews with Latino caregivers of PWD and caregiver advocates. We recruited both from community organizations, senior centers, and clinics. Our interview guide focused on experiences of caregiving, interactions with medical system, and knowledge and experiences managing behavioral and eating problems. We used Grounded Theory methodology for coding and analysis, focusing on contrasting and comparing experiences within and between caregivers and caregiver advocates. RESULTS/ANTICIPATED RESULTS: Preliminary results from interviews with two caregivers and two caregiver advocates illustrate that caregivers of persons with dementia have a difficult time receiving high quality care from primary care clinicians. All participants noted that many primary care doctors didn’t know how to diagnose ADRD and dismissed critical symptoms as part of old age. Caregivers also reported that they wished they had more information on what to expect with ADRD disease progression, noting they received little information from the formal medical care system. With respect to behavioral problems, caregiver advocates noted that primary care doctors often did not provide non-pharmacological alternatives to behavioral problems. DISCUSSION/SIGNIFICANCE OF FINDINGS: Findings from our pilot study demonstrate that there is a clear need to train primary care physicians who serve Latino older adults on ADRD care. Improved diagnosis and management could improve outcomes among Latino older adults with dementia.

